# Real life management of community-acquired Pneumonia in adults in the Gulf region and comparison with practice guidelines: a prospective study

**DOI:** 10.1186/s12890-015-0108-x

**Published:** 2015-09-30

**Authors:** Bassam Mahboub, Ashraf Al Zaabi, Ola Mohamed Al Ali, Raees Ahmed, Michael S. Niederman, Rania El-Bishbishi

**Affiliations:** Rashid Hospital, Oud Metha Road, Umm Hurair Area 2, PO Box 4545, Dubai, UAE; Zayed Military Hospital, Abu Dhabi, United Arab Emirates; Rashid Hospital Trauma Center, Dubai, United Arab Emirates; Winthrop-University Hospital, Mineola, USA; Department of Medical Affairs, Sanofi, Dubai, United Arab Emirates

**Keywords:** Community-acquired pneumonia, Management, Guidelines, Gulf countries

## Abstract

**Background:**

Very few data exist on the management of community-acquired pneumonia (CAP) in patients admitted to hospitals in the Gulf region. The objectives of this study were to describe treatment patterns for CAP in 38 hospitals in five Gulf countries (United Arab Emirates, Kuwait, Bahrain, Oman, and Qatar) and to compare the findings to the most recent Infectious Diseases Society of America (IDSA) / American Thoracic Society (ATS) guidelines.

**Methods:**

This was a prospective, observational study conducted between January 2009 and February 2011. Adult patients hospitalised (excluding intensive care units) for CAP and subsequently discharged were included. Data were collected retrospectively at hospital discharge, and prospectively during two follow-up visits. Data on medical history, mortality-risk scores, diagnostic criteria, antibiotic treatment, isolated pathogens and clinical and radiographic outcomes were collected. Care practices were compared to the IDSA/ATS guidelines.

**Results:**

A total of 684 patients were included. The majority (82.9 %) of patients were classified as low risk for mortality (pneumonia severity index II and III). The majority of patients fulfilled criteria for treatment success at discharge, although only 77.6 % presented a normalised leukocyte count. Overall, the management of CAP in Gulf countries is in line with the IDSA/ATS guidelines. This applied to the diagnosis of CAP, to the identification of high-risk CAP patients, to the identification of etiologic agent responsible for CAP and to the type of treatment despite the fact that combinations of antimicrobial agents were not consistent with the guidelines in 10 % of patients. In all patients, information about Gram’s staining was not captured as recommended by the IDSA/ATS and in the majority of patients (>85 %) chest radiography was not systematically performed at the post-discharge follow-up visits.

**Discussion:**

The management of CAP in the Gulf region is globally in line with current IDSA/ATS guidelines, although rates of pathogen characterisation and post-discharge follow-up need to be improved.

**Conclusion:**

Compliance with established guidelines should be encouraged in order to improve the management of the disease in this region.

## Background

Community-acquired pneumonia (CAP) is a common and potentially serious illness acquired outside a hospital or long-term care facility or other recent contact with the health care system [1].

Improving the care of adult patients with CAP has been the focus of many different organisations, and several have developed guidelines for the treatment of CAP. In North America, CAP guidelines were first developed in 1993, and in 2007, the joint Infectious Diseases Society of America (IDSA) / American Thoracic Society (ATS) guidelines [2, 3] were proposed. The process, which began in the United States and Canada, has now been implemented in numerous countries throughout the world [4]. In the Gulf region, the IDSA/ATS guidelines are considered as one of the most important practice guidelines for the diagnosis and management of CAP. Previous studies have shown that implementing IDSA/ATS guidelines for hospitalised CAP patients in general wards is associated with good patient outcomes [5–7]. For instance, a study conducted in the United States has evaluated the impact of use of the IDSA/ATS therapeutic guidelines among 35 477 inpatients with CAP. This study showed that, after adjustment for severity of illness and other potential confounders, guideline-concordant therapy was associated with a decrease of in-hospital mortality (odds ratio [OR], 0.70; 95 % confidence interval [CI], 0.63–0.77) and of sepsis (OR, 0.83; 95 % CI, 0.72–0.96), with a reduction in both length of stay and duration of parenteral therapy by approximately 0.6 days (*p* < 0.001 for both comparisons) [5]. Several studies on the outcomes of patients with CAP and adherence to the 2007 IDSA/ATS guidelines [2] have been published [8–10]. However, to our knowledge, only one published study in a Gulf Arab state (Oman) [11] has been conducted to compare the inpatient management of CAP with established clinical guidelines (the Gulf Cooperation Council CAP guidelines (GCC CAP) [12]).

The main objectives of our study were to describe treatment practices in CAP patients admitted to hospitals (but not to intensive care units) in five countries in the Gulf region (United Arab Emirates, Kuwait, Bahrain, Oman and Qatar) and to compare the findings to the 2007 IDSA/ATS treatment guidelines.

## Methods

### Study design

The Gulf practices of Treatment of in-hospital Community-Acquired Pneumonia (G-TinCAP) study was a multicentre, prospective, observational study conducted in 38 hospitals. All these were large urban hospitals involved in the management of CAP and who admitted adequate number of CAP patients. These hospitals included both public and private institutions, teaching hospitals, community hospitals and military hospitals. A total of 21 centres from United Arab Emirates, ten centres from Kuwait, three centres from Bahrain, three centres from Oman, and one centre from Qatar were involved. In principle, these hospitals adhered to the IDSA/ATS guidelines [2] for management of infectious diseases published in 2007, as well as local Gulf Cooperation Council CAP guidelines [12]. The study was conducted between January 2009 and February 2011.

### Study population

Participating investigators were selected from general practitioners, internists and pulmonologists practicing in hospitals from five Gulf countries. Patients who completed a hospitalisation for CAP resulting in discharge were eligible for the study. Adult patients (aged over 18 years) diagnosed with a CAP class II, ІІІ, ІV or V according to the Fine score criteria of severity [13], were included in the study if they provided a signed informed consent.

The diagnosis of CAP was based on:

- At least one of the following symptoms:
  1. Presence of fever (pyrexia);
  2. Shivering;
  3. Presence of severe cough (with or without expectoration) or persistent cough with discolored phlegm; pleuritic chest pain or dyspnea.

b) Presence of auscultation findings of pneumonia namely crepitations or later inspiratory crackles in involved areas and bronchial breathing.
c) Chest radiograph with evidence of new infiltrate changes compatible with CAP (not required for all patients).

The exclusion criteria consisted of:

- Participation in another clinical trial;
- Patients with CAP categorised in class І according to Fine criteria [13], or in any class, who required treatment in an Intensive Care Unit (ICU) at any time;
- Patients previously hospitalised for any reason within 15 days prior to the current hospitalisation;
- Patients with lung cancer, proven tuberculosis or HIV

Individual patients who were admitted on more than one occasion with a CAP could only be included once.

The analysis was performed on all patients discharged from hospital.

### Data collection

Elligible patients who agreed to participate in the study were invited to take part in the study and a case report form was completed at discharge from hospital, during the first follow-up visit between 7 and 10 days after discharge, and during a final follow-up made either by telephone or at a hospital visit around 3 weeks after discharge by the investigator. Data on patient’s socio-demographic characteristics, medical history, diagnosis, comorbidities and treatment were collected. Specific information on the CAP was also collected including initial evaluation of the CAP, risk class according to the Fine criteria [13]; clinical and radiographic symptoms of CAP as well as evidence of pulmonary infiltrates compatible with CAP and bacteriological evaluation. Concomitant medications and antimicrobial treatments up to 14 days before study entry were also documented.

Data were validated for quality by checking for completeness, discrepancies, and inconsistencies, which, if present, were queried to the investigators for clarification before the data were validated in the database.

### Study outcomes

Data regarding the management of CAP in the Gulf countries was documented and compared with treatment recommendations of the IDSA/ATS guidelines published in 2007. Overall, CAP diagnosis criteria, as well as criteria for the identification of high-risk patients, tests performed for the identification of aetiological agent responsible for CAP and the treatment used were described and compared with the international guidelines.

The overall therapeutic outcome on CAP (success or failure) is documented by participating physicians at discharge visit and subsequent follow up visits. The therapeutic outcome was considered successful if the “patient’s clinical stability at discharge” criteria had been achieved. These were stable vital signs over 24 h, ability to take oral medication, normal eating and drinking behavior, return to usual mental state, and normalisation of leukocyte count. The therapeutic outcome was considered as failure in case of development of complications such as pulmonary abscess or sepsis, progression of clinical symptoms, radiological changes, need to change the antibiotic therapy, rehospitalisation due to pneumonia, and death. The switch from intravenous to oral administration was also evaluated according to four criteria, consisting of patient’s ability to eat and drink, negative hemocultures, temperature ≤38°С (during the last 16 h) and improvement of symptoms. Fever and leukocytosis were not considered as a reason for switch.

### Statistical analysis

The sample size was determined *a priori* assuming that 50 % of hospitalised CAP patients are high-risk patients (pneumonia severity index (PSI) IV, V), as reported in a previous study by Etzion et al.[14]. In order to estimate a proportion of high-risk patients of 50 % with a 95 % confidence interval of ±4 %, it was estimated that a target sample of 583 patients would be required to be included, which was rounded up to 600 patients. Assuming a dropout rate of 15 %, about 690 patients were planned to be recruited for this study. All enrolled patients were included in the final analysis, which was performed on data pooled from all five participating countries. Data were summarized using descriptive statistics including arithmetic means, ranges, frequencies and percentages. Statistical analysis was performed using SPSS software, version 16 (SPSS Inc., Chicago, USA).

### Ethics

The study was performed in accordance with the Guidelines of Good Epidemiology Practice [15] and corresponding local regulations, including data confidentiality. This study was approved by the Institutional Review Boards of all hospitals when required according to local regulations and a written informed consent was obtained from all eligible patients.

## Results

### Characteristics of the study population

A total of 684 patients were enrolled in the study by 39 physicians from 38 centres located in the five participating countries. All participating subjects met eligibility criteria of the study and no major protocol deviations were identified during the study. Of the 684 patients, 444 were from the United Arab Emirates, 150 patients were from Kuwait, 44 from Bahrain, 28 from Oman and 18 from Qatar. The baseline characteristics of participating patients are presented in Table 1.

**Table 1:** Baseline characteristics of the study population

	Total
	(*N* = 684)
Gender (men)	505 (73.8 %)
Age (years)	41.5 ± 14
Living in own home	464 (67.8 %)
Living in a skilled nursing facility	220 (32.2 %)
Current smoker	316 (46.2 %)
Imuunocompetent	669 (97.8 %)
Principal CAP associated disease	
Cardiovascular disease	71 (10.4 %)
Diabetes mellitus	85 (12.4 %)
Bronchopulmonary disease	11 (1.6 %)
Renal failure	13 (1.9 %)
Chronic liver disease	11 (1.6 %)

Data are presented as numbers of patients (%) except for age, which is presented as mean ± SD. CAP indicates community-acquired pneumonia

The classification of CAP risk according to the Fine criteria [13], as recommended in the IDSA/ATS guidelines, is presented in Table 2. The majority (82.9 %) of patients were classified as low risk for mortality [13] (classes II and III). During chest auscultation, positive clinical findings such as crepitations were documented by 80 % of study participants. At admission, 70.6 % (*n* = 483) of the patients showed new infiltrative changes in the chest radiogram; for the rest of the population, a radiogram was not performed or did not show new infiltrative changes. In addition, 12.1 % (*n* = 83) of patients received antibiotic treatment prior to hospital admission (Table 2).

**Table 2:** Baseline risk classification, diagnostic criteria of CAP and pre-study use of antibiotics

	*N* = 684
FINE criteria [13]	
Mean score [range]	69 ± 27 [18–167]
Category II	386 (56.4 %)
Category III	181 (26.5 %)
Category IV	100 (14.6 %)
Category V	17 (2.5 %)
Clinical and radiologic CAP criteria	*N* = 684
Pyrexia	676 (98.8 %)
Shivering	631 (91.9 %)
Severe cough or persistent cough with discolored phlegm	682 (99.7 %)
Crepitation/late inspiratory crackles or bronchial breathing	553 (80.9 %)
Chest radiograph (X-ray)	483 (70.6 %)^a^
Antibiotic therapy used 14 days prior to study entry	*N* = 83
Co-amoxiclav	23 (27.7 %)
Cefuroxime	16 (19.3 %)
Clarithromycin	15 (18.1 %)
Amoxycillin	10 (12.1 %)
Ceftriaxone	7 (8.4 %)
Azithromycin	5 (6.0 %)
Levofloxacin	3 (3.6 %)
Cefixime	2 (2.4 %)
Moxifloxacin	1 (1.2 %)
Penicillin	1 (1.2 %)

Data are presented as numbers of patients (%) unless otherwise indicated. *CAP* community-acquired pneumonia

^a^Only patients with new infiltrative changes in the radiogram of the chest

Pathogen isolation was performed in 88 patients. Only 78 specimens collected (mainly sputum) were analyseable, of which *Streptococcus pneumonia* was present in almost half of them (46.1 %; *n* = 36 specimens), followed by the ccombination of *S. pneumoniae* and *Klebsiella* which was isolated in 17 % of cases (*n* = 13 specimens), and *Klebsiella* alone and *Mycoplasma* alone which were isolated in 9 % (*n* = 7 specimens) and 8 % (*n* = 6 specimens) of cases respectively.

### Empirical therapy and process of care

The mean length of hospital stay for the treatment of CAP was 3.9 ± 4.4 days ranging from 1 to 19 days. For all study participants, intravenous empirical antibiotic treatment was initiated immediately upon hospital admission, before the pathogen was identified. The mean duration of intravenous treatment was 3.5 days (81 h), ranging from 3 to 6 days. In 80 % of patients, switching to oral antibiotic therapy occurred within 3 days (72 h). The physicians’ decision to switch was mainly driven by lack of improvement of basic clinical CAP symptoms.

The main factor determining the choice of CAP antimicrobial therapy was the type of previous antibiotic therapy, reported in 406 (59.4 %) patients, followed by interaction with other medicines, reported in 285 (41.7 %) patients. In the vast majority of patients, (94.4 %; *n* = 646), antimicrobial therapy initiated on the day of hospitalisation was not changed. The remaining patients (*n* = 38) required changes from one class of antibiotic to another, most frequently within the 24 h following the isolation of organism, based on the sensitivity of pathogens to particular antimicrobial agents.

The majority of patients (77.9 %, *n* = 533) were treated with a single antimicrobial agent, whereas about one fifth of patients (20.3 %; *n* = 139) were treated with two agents and the rest (1.8 %; *n* = 12) with three agents. In the group of patients treated with a single antimicrobial agent, levofloxacin was the most frequently used (65.7 %; *n* = 350), followed by ceftriaxone (16.1 %; *n* = 86) and moxifloxacin (13.1 %; *n* = 70). Other single antibiotics, such as co-amoxiclav, were used in less than 2 % of patients. Use of the most frequent combinations of two antimicrobial agents is shown in Table 3. Levofloxacin was most frequently used in combinations with other antibiotics (57.7 % of cases), principally with a β-lactam (50.5 % of cases, corresponding to around 10 % of the entire study population). At discharge, most patients fulfilled the criteria for treatment success. The determination of success was based on patient’s clinical stability and discharge criteria. Thus, vital signs were stable over 24 h in 99.3 % of patients, 99.7 % were able to take oral antibiotics, 99.3 % were able to eat and drink normally, 94.1 % were evaluated as being in their normal mental condition and leukocyte count had normalised in 77.6 % of patients. At the time of discharge, all patients underwent a chest radiogram, although it should be noted that complete radiographic recovery had only occurred in 31.4 % (*n* = 215 patients) of patient.

**Table 3:** Frequency distribution and patterns of combination of two antimicrobial agents in hospitalised CAP patients (Total *N* = 139)

	Levofloxacin	Ceftriaxone	Azithromycin	Tazocin
Levofloxacin		-	-	-
Ceftriaxone	31.7 %		-	-
Azithromycin	0.7 %	13.7 %		-
Tazocin	9.4 %	-	7.2 %	
Co-amoxiclav	2.9 %	-	8.6 %	-
Clarithromycin	2.2 %	4.3 %	-	0.7 %
Cefipime	3.6 %	-	-	-
Amikacin	2.9 %	-	-	-
Cefuroxime	2.9 %		-	-
Moxifloxacin	-	2.2 %	-	2.9 %
Ciprofloxacin	-	-	0.7 %	-
Co-trimoxazole	0.7 %	-	-	-
Gentamycin	0.7 %	-	-	-

At hospital discharge, a total of 626 patients (91.5 %) were prescribed home antimicrobial therapy, most frequently oral therapy unless the patient could not tolerate this route of administration. Of these, 573 patients (91.5 %) used a single antimicrobial agent and 53 (8.5 %) used a combination of two agents. The mean duration of treatment was 7.4 days [6–10 days] and 75 % of patients were prescribed a home antibiotic for 5–10 days. Among the recorded single antimicrobial agents in 519 patients, levofloxacin was used in the majority of patients (62.6 %; *n* = 325), followed by moxifloxacin (13.5 %; *n* = 70), co-amoxiclav (11.2 %; *n* = 58), cefuroxime (6.5 %; *n* = 34), and clarithromycin (2.7 %; *n* = 14). Among the recorded combination therapies in 47 patients, levofloxacin plus cefuroxime was the most frequently used (34.0 %; *n* = 16) followed by levofloxacin plus co-amoxiclav (19.1 %; *n* = 9).

The first follow-up visit was performed for the entire study population (*n* = 684) and took place after a mean of 9 days after hospital discharge. The second follow-up consultation was performed for 27.3 % (*n* = 187 patients) and took place after a mean of 11 days after first follow-up. Data was collected either by telephone (63.6 %; *n* = 119,) or during a hospital visit (36.4 %; *n* = 68).

During the two follow-up visits, participating physicians considered that the treatment was successful in almost all patients (>99.5 %) and adherence to CAP therapy at home was reported by almost all patients (>95.0 %) as well. However, radiographic assessment was not performed in the majority of patients (>85 %), so that this could not be used as a criterion for success. Clinical CAP symptoms and signs were present in 25.4 % (*n* = 174) and in 2.0 % (*n* = 3) of the patients respectively during the first and second follow-up visits.

### Comparison of medical practices in the Gulf region with CAP guidelines

The principal recommendations issued by the IDSA/ATS guidelines are presented in Table 4, together with the corresponding practice in Gulf countries as documented in the current study. The IDSA/ATS recommends that for CAP cases, baseline history should include considerations such as cough, fever, and previous hospitalisation for pneumonia, as well as additional contributing risk factors. The study revealed that cough (acute or changed character of chronic cough) was found in 99 % of cases while pyrexia was observed in 98 % of CAP cases. During chest auscultation, positive clinical findings (crepitations, late inspiratory crackles or bronchial breathing) were documented in 80 % of study participants.

**Table 4:** The IDSA/ATS guidelines and corresponding practices in the Gulf region documented by the G-TinCAP study

IDSA/ATS steps guide [2]	IDSA/ATS recommendations [2]	Gulf practices according to G-TinCAP study
Make a correct diagnostic of CAP	Consider cough, fever, and previous hospitalization for pneumonia.	The study captured the information about cough and fever as one of the clinical criteria for CAP.
	Additional contributing risk factors for CAP include viral infections, neutropenia, pulmonary edema, altered consciousness, airway obstruction, and congenital pulmonary abnormalities.	Accompanying diseases for vital organs and other important information (eg malignancy, smoking and immunocompetency) were included in the baseline medical history.
Confirm the diagnosis	Chest radiography should be carried out for all suspected CAP patients to confirm the presence of pneumonia.	Chest auscultation was performed to detect any positive clinical findings (Crepitations, late inspiratory crackles or bronchial breathing).
		70.6 % (*n* = 483) of the patient had new infiltrative changes in the radiogram of the chest. For the rest of the population radiogram was not performed or was not showing new infiltrative changes.
Identify high-risk CAP patients	Use IDSA/ATS algorithm to define which patients would benefit from hospitalisation.	The Fine criteria [13], which use all the parameters included in IDSA/ATS algorithm, were documented for all patients in this study.
Identify aetiologic agent responsible for CAP	*Streptococcus pneumoniae* is the most common etiologic agent for CAP.	*Streptococcus pneumoniae* alone was isolated in 46 % of cases (*n* = 36 specimens). The combination of *S. pneumoniae* and *Klebsiella* was isolated in 17 % of cases (*n* = 13 specimens). *Klebsiella* alone and *Mycoplasma* alone were isolated in 9 % (*n* = 7 specimens) and 8 % (*n* = 6 specimens) of cases respectively. *S. viridans*, *Moraxella cararrhalis* and *Legionella* were isolated in only 4 % of cases (*n* = 3 specimens)*.*
	*Haemophilus influenzae* and *Chlamydia pneumoniae* are generally the third or fourth most common organisms identified. Less common etiologic agents include oral anaerobes, Staphylococcus aureus, Legionella pneumophila and Moraxella catarrhalis.	
Establish the aetiology and ensure that the therapy is pathogen directed	Use of sputum Gram’s stain and culture in all patients, whenever possible.	Information about Gram’s staining was not captured, but data was collected for the type of specimen used for isolation along with the type of test requested. Sputum was the specimen most frequently used.
Antimicrobial therapy	Empiric antimicrobial therapy should be initiated until laboratory results can be obtained to guide more specific therapy.	In all participants, empirical antimicrobial therapy was initiated immediately on the day of hospitalisation without waiting the laboratory results (culture and sensitivity tests).
	Either fluoroquinolones or macrolides plus doxycycline are suggested for primary empiric therapy.	Levofloxacin or moxifloxacin (fluoroquinolones) were the most frequently used antimicrobial agents during hospital stay and after hospital discharge.
Route of administration for the antibiotic therapy	Switch to an appropriate oral antibiotic is recommended as soon as the patient’s condition is stable and he or she can tolerate oral therapy, often within 72 h.	The mean duration of intravenous administration of CAP therapy was around 81 h, though in 80 % of cases, switch to an oral antibiotic occurred within 3 days (72 h).
Treatment duration	As far as duration of treatment is concerned, the treatment for *S. pneumoniae* should generally continue for 7 to 14 days or until the patient is afebrile for 72 h.	During hospitalisation, the mean duration of treatment with antimicrobial agents was 3.5 days, ranging from 3 to 6 days.
	Patients with atypical pathogens should be treated for 10 to 21 days.	After hospital discharge, the mean duration of treatment with antimicrobial agents was 7.4 days, ranging from 6 to 10 days.
		Over 80 % of patients were prescribed a single antimicrobial agents for their home antibiotic therapy after discharge from hospital.

As recommended by the IDSA/ATS guidelines, in 70 % (*n* = 483) of patients a chest radiograph was indicative of new infiltrative changes. Moreover, to identify high risk patients requiring hospitalisation, our study reported that Gulf countries used the Fine criteria [13], which is one of the instruments suggested in the IDSA/ATS guidelines to identify patients with CAP who may be candidates for outpatient treatment. In addition, the most common CAP aetiological pathogens in the study was *S. pneumoniae*, which is in line with the observations of the IDSA/ATS. However, the number of isolates was limited (*n* = 78). Information about Gram’s staining was not captured as recommended by the IDSA/ATS, but data regarding the type of specimen used for isolation of organism along with the type of test requested were collected. Empirical antimicrobial therapy was initiated immediately on the day of hospitalisation without waiting for culture and sensitivity results, which is not the recommended option. Moreover, it was reported that in almost 95 % of subjects empirical antimicrobial therapy was not changed after the isolation of organism. The most frequent primary empiric therapy used was levofloxacin (fluoroquinolones), which is consistent with the IDSA/ATS guidelines.

## Discussion

This study was a multicentre, multinational, observational study which aimed to describe treatment practices in CAP patients admitted to hospitals in five countries in the Gulf region (United Arab Emirates, Kuwait, Bahrain, Oman and Qatar) and to compare the findings to the 2007 IDSA/ATS treatment guidelines. To our knowledge, this is one of the first studies to assess treatment standards, which has included a large sample of patients to be conducted in the Gulf region.

Overall, our study shows that the management of CAP in Gulf Arab countries participating in the study is in line with the IDSA/ATS guidelines. For example, information on cough and fever as one of the clinical criteria for CAP was collected systematically and chest radiography showed new infiltrative changes in the majority of patients (70.6 %).

However, for the rest of the population, a radiogram was not performed or did not show new infiltrative changes.

The type, route of administration and duration of antimicrobial therapy was also generally consistent with the IDSA/ATS guidelines for hospital ward treatment. Antimicrobial therapy used in the Gulf region was in general consistent with the recommendation of starting initial therapy empirically with fluoroquinolones until laboratory results could be obtained to guide more specific therapy. All patients received therapy initiated immediately on the day of hospitalisation, with fluoroquinolones being the most widely used treatment. However, ten percent of the study population received a combination of a fluoroquinolone and a β-lactam during hospital admission, which is not the treatment recommended by the IDSA/ATS expert panel for patients not admitted to an ICU [2].

The pathogens responsible were not isolated and characterised in the majority of patients and thus represent the major divergence between current practice in the Gulf region and the IDSA/ATS guidelines. Characterisation of CAP pathogens was only achieved in 12 % of patients, principally by analysis of sputum samples. In addition, information about use of Gram’s staining method or any other methods used to identify pathogens, was not collected in our study. The most frequently identified pathogen was *Streptococcus pneumoniae*, which is consistent with findings from other countries, as noted in the IDSA/ATS guidelines [2].

In 95 % of cases, the initial empiric therapy in our study was not switched, suggesting that this empiric therapy was effective. However, since the responsible pathogen was not isolated in the majority of the patients, it is not known whether the treatment was the most appropriate in these patients. Once the patient was clinically stable, around 80 % of patients were switched to an oral antibiotic within three days (72 h) as recommended in the guidelines.

A further objective was to evaluate treatment outcome at hospital discharge and up to approximately 3 weeks after discharge. At hospital discharge, criteria for treatment success were fulfilled by the majority of patients. However, around one quarter of patients were discharged before their leukocyte count had normalised. In addition, it should be noted that at hospital discharge, complete radiographic recovery was only detected in around one-third of patients. Clinical signs related to CAP were resolved in 97.9 % (*n* = 183) at the last follow-up visit, performed around 3 weeks after discharge.

Our study identifies four areas in which current practice for management of CAP in the Gulf region falls short of international guidelines and could be improved.

The first corresponds to isolation and characterisation of the pathogenic agent, which needs to be performed systematically. The lack of pathogen isolation in the majority of patients observed in the study may result in inappropriate antimicrobial therapy being prescribed and thus expose patients to unnecessary risk.

The second relates to the failure to perform chest radiography systematically at the post-discharge follow-up visits, with around 90 % of patients not being assessed. This may result in residual infection in the lungs being missed and increase the risk of relapse if the physician or patient decides to interrupt antimicrobial treatment. Nonetheless, in everyday clinical practice in North American and Europe, it is also common for follow-up chest radiographs to be performed only if symptoms persist or there is a clinical suspicion of infection [16].

The third relates to the fact that only one quarter of patients attended a second follow-up visit 3 weeks after discharge. It would be important to encourage physicians to collect information from their patients, either during a formal hospital visit, and particularly if residual clinical or radiological signs of infection were detected at the previous follow-up visit, or by telephone, 3 weeks after discharge to ensure that the patient is effectively cured.

Finally, 10 % of the study population received a combination of a fluoroquinolone and a β-lactam during hospital admission, which is the treatment recommended by the IDSA/ATS expert panel for patients directly admitted to an ICU [2] and not for patients included in our study (*ie* patients not directly admitted to ICU). Consequently, in those patients the choice of CAP antimicrobial therapy was not consistent with the guidelines. Neverthless, at hospital admission, seventeen percent of patients (*N* = 117) were classified as moderate to high risk for mortality according to the Fine criteria [13] and consequently could be considered by the physician to be eligible for an ICU regimen without necessarily being directed to one. Because the overall efficacy of many classes of antimicrobial regimens is considered to be good, over-treatment use is expected to produce the same clinical outcome but with an increased risk of antiobiotic resistance and side effects. For this reason, the selection of antimicrobial regimens for empirical therapy should be based on prediction of the most likely pathogen and knowledge of local susceptibility patterns with a preference given to the more potent drugs because of their benefit in drecreasing the risk of selection for antiobiotic resistance [2].

We compared treatment practices in CAP patients in the Gulf region to the IDSA/ATS guidelines since they are the most used guidelines worldwide, are well known and used by doctors of the region and are one of the recommended standard of care in the Gulf region.

The adherence to regional guidelines (*ie* GCC CAP guidelines [12]) has already been evaluated in Oman [11]. This analysis showed that there was very poor adherence to the guidelines with respect to CAP severity assessment and provision of preventive measures upon hospital discharge and highlighted the necessity to improve the implementation of such guidelines in the region. Several studies have evaluated the level of adherence to the IDSA/ATS guidelines. For instance, a recent study from Italy performed in 191 patients with CAP revealed that the adherence to antibiotic treatment guidelines was poor, since only 47 % of patients received an empirical antibiotic regimen that was adherent to guidelines [17].

The enrolled patients were principally at low mortality risk (82.9 %) according to the Pneumonia Severety Index (PSI) developed by Fine [13], which assigns points based on age, laboratory parameters, and the presence of different comorbidities. The PSI is one of the instruments recommended by the IDSA/ATS panel in order to assess mortality risk and to identify patients with CAP who may be candidates for outpatient treatment [2]. It has been suggested that patients with risk class I and II should be treated as outpatients, risk class III patients should be treated in an observation unit or with a short hospitalisation, and risk class IV and V patients should be treated as inpatients [13]. In the present analysis, we decided not to include patients with risk class I. We assumed that those patients did not require a particular hospital monitoring and are most often candidates for outpatient treatment than that of other classes.

The proportion of patients considered at low mortality risk (PSI II and III) was higher than that anticipated and also higher than the rate reported in the literature. For example, it has been reported that the rate of low-risk patients hospitalised due to CAP in Israel, USA and Western Europe was around 30 %[13, 14, 18, 19]. The most likely explanation for this is that patients at high risk, who in fact died during hospitalisation, are not captured in our study.

A principal limitation of our study is the lack of information on methods used for pathogen identification and the failure to characterise the pathogen in the majority of patients, which compromises the quality of the data collected. In particular, this may have led to less frequent, but epidemiologically important, pathogens not being detected.

A second major limitation relates to the mode of recruitment of the patients, which precludes collecting information on in-hospital mortality. We were thus unable to document mortality rates in our cohort, in particular in patients with moderate to high risk mortality according to the Fine criteria [13]. According to the literature, the rate of mortality has been estimated to be low in the Gulf region compared to other areas of the world. For instance, the mortality rate among patients with pneumoniae has been estimated at 3.5 % (*N* = 6/172 patients) in Oman [11] compared to 7.2 % (*N* = 147/ 2039 patients) in Europe [20].

A third limitation relates to the low mortality risk of the majority of the patients enrolled. This may be a consequence of exclusion of patients who required hospitalisation in an ICU and failure to capture high risk patients who died in hospital. In the sample size calculation, it was anticipated that 50 % of patients would be at high risk, whereas in fact >80 % of patients were at low risk. This enrichment in low-risk patients may have led to an over-optimistic view of treatment outcome in clinical practice, which may not apply to more severely-ill patients. In addition, it is possible that standards of care, in terms of adherence with IDSA/ATS guidelines, may have been inferior in patients who died.

Finally, although we examined data from five Gulf countries represented by 38 hospitals, the majority of the study population (65 %; *n* = 444) came from the United Arab Emirates, which may introduce some bias. To a certain extent, the number of patients included reflects the demographic weight of each country, with UAE having the highest population (8 million) in 2010, followed by Kuwait and Oman (3 million each), Qatar (1.8 million) and Bahrain (1.2 million) [21]. However, the UAE is somewhat over-represented and Qatar and Oman somewhat under-represented. In part this may be explained by the number of hospitals available for recruitment. The federal structure of the UAE has led to the development of multiple autonomous health services in each of the constituant emirates leading to a larger number of hospitals *per capita* than in the other Gulf countries. In contrast, in Oman and Qatar, the population, and health care provision, is concentrated in the capital city. The number of patients recruited in Oman was particularly low (28). In comparison, a study performed only in Oman [11] in a single centre and over a similar study period to that of our study (2 years), included a total of 170 patients. It is possible that there was competition for patient recruitment between the two studies. Moreover, all participating countries have large populations of migrant workers whose socioeconomic status differs from the local population. Socioeconomic status may influence morbidity risk and adherence with treatment following discharge and this was not taken into account in our analysis. Finally, it should be noted that our study did not include Saudi Arabia, which is the largest country of the Gulf region. Consequently, our results should not be generalised to the entire Gulf region.

## Conclusion

This study provides a valuable “snapshot“ of current CAP treatment practices in the Gulf region and reveals that the management of CAP in this region is generally in line with current IDSA/ATS guidelines, although rates of pathogen characterisation and post-discharge follow-up need to be improved. This study highlights the importance of improving the implementation of the established guidelines in order to improve outcome of patients with CAP in the Gulf region.

## References

[1] Wunderink RG, Waterer GW (2014). Clinical practice. Community-acquired pneumonia. N Engl J Med.

[2] Mandell LA, Wunderink RG, Anzueto A, Bartlett JG, Campbell GD, Dean NC (2007). Infectious Diseases Society of America/American Thoracic Society consensus guidelines on the management of community-acquired pneumonia in adults. Clin Infect Dis.

[3] Bernstein JM (1999). Treatment of community-acquired pneumonia--IDSA guidelines. Infectious Diseases Society of America. Chest.

[4] Niederman MS, Luna CM (2012). Community-acquired pneumonia guidelines: a global perspective. Semin Respir Crit Care Med.

[5] McCabe C, Kirchner C, Zhang H, Daley J, Fisman DN (2009). Guideline-concordant therapy and reduced mortality and length of stay in adults with community-acquired pneumonia: playing by the rules. Arch Intern Med.

[6] Grenier C, Pepin J, Nault V, Howson J, Fournier X, Poirier MS (2011). Impact of guideline-consistent therapy on outcome of patients with healthcare-associated and community-acquired pneumonia. J Antimicrob Chemother.

[7] Watkins RR, Lemonovich TL (2011). Diagnosis and management of community-acquired pneumonia in adults. Am Fam Physician.

[8] Arnold FW, LaJoie AS, Brock GN, Peyrani P, Rello J, Menendez R (2009). Improving outcomes in elderly patients with community-acquired pneumonia by adhering to national guidelines: Community-Acquired Pneumonia Organization International cohort study results. Arch Intern Med.

[9] Ramirez J (2005). Multicenter, multinational observational studies: a new approach to studying community-acquired pneumonia. Respir Care Clin N Am.

[10] Fahimi F, Moradi M, Safarnavadeh T, Namdar R (2007). Management of Community Acquired Pneumonia in a Teaching Hospital: The Role of Established Guidelines. Tanaffos.

[11] Al-Abri SS, Al-Maashani S, Memish ZA, Beeching NJ (2012). An audit of inpatient management of community-acquired pneumonia in Oman: a comparison with regional clinical guidelines. J Infect Public Health.

[12] Memish ZA, Arabi YM, Ahmed QA, Shibl AM, Niederman MS (2007). Management and prevention strategies for community-acquired pneumonia in the Gulf Corporation Council. J Chemother.

[13] Fine MJ, Auble TE, Yealy DM, Hanusa BH, Weissfeld LA, Singer DE (1997). A prediction rule to identify low-risk patients with community-acquired pneumonia. N Engl J Med.

[14] Etzion O, Novack V, Avnon L, Porath A, Dagan E, Riesenberg K (2007). Characteristics of low-risk patients hospitalized with community-acquired pneumonia. Eur J Intern Med.

[15] Andrews EA, Avorn J, Bortnichak EA, Chen R, Dai WS, Dieck GS (1996). Guidelines for Good Epidemiology Practices for drug, device, and vaccine research in the United States. Pharmacoepidemiol Drug Saf.

[16] Patterson CM, Loebinger MR (2012). Community acquired pneumonia: assessment and treatment. Clin Med.

[17] Rossio R, Franchi C, Ardoino I, Djade CD, Tettamanti M, Pasina L, et al. Adherence to antibiotic treatment guidelines and outcomes in the hospitalized elderly with different types of pneumonia. Eur J Intern Med. 2015. 10.1016/j.ejim.2015.04.002 25898778

[18] Lim WS, Macfarlane JT, Boswell TC, Harrison TG, Rose D, Leinonen M (2001). Study of community acquired pneumonia aetiology (SCAPA) in adults admitted to hospital: implications for management guidelines. Thorax.

[19] Ewig S, Torres A, Woodhead M (2006). Assessment of pneumonia severity: a European perspective. Eur Respir J.

[20] Blasi F, Garau J, Medina J, Avila M, McBride K, Ostermann H (2013). Current management of patients hospitalized with community-acquired pneumonia across Europe: outcomes from REACH. Respir Res.

[21] United Nations Departement of Economic and Social Affairs (2015). Population Division.

